# Subsequent management and outcomes after first-line PARP inhibitors progression in ovarian cancer patients

**DOI:** 10.1186/s13048-024-01400-9

**Published:** 2024-04-01

**Authors:** Hua Yuan, Ning Li, Lingying Wu, Hongwen Yao

**Affiliations:** Department of Gynecologic Oncology, National Cancer Center/National Clinical Research Center for Cancer/Cancer Hospital, Chinese Academy of Medical Sciences and Peking Union Medical College, Beijing, 100021 China

**Keywords:** Subsequent management, Outcome, First-line PARPi progression, Ovarian cancer

## Abstract

**Objectives:**

This retrospective study aims to evaluating the subsequent management and outcomes after first-line PARPi progression in Chinese ovarian cancer population.

**Methods:**

Clinical and pathologic variables, treatment modalities, and outcomes were assessed. We investigated the subsequent management and outcomes after first-line PARPi progression. The objective response rate (ORR) and disease control rate (DCR) parameters were evaluated to determine the response to subsequent chemotherapy. For the survival analyses, progression-free survival 1 (PFS1), PFS2, overall survival (OS) and PFS2 − PFS1 were analysed.

**Results:**

A total of 124 patients received PARPi maintenance treatment after first-line chemotherapy during the study period in our center. 44 of them (35.5%) experienced a recurrence. The median duration of PARPi in these patients was 11.1 months (range: 1.2–75.1 months). A total of 40 patients (40/44, 90.9%) received subsequent chemotherapy with 35 (35/44, 79.5%) and 5 (5/44, 11.4%) patients received platinum-based and non-platinum-based chemotherapy in our center. 2 patients (4.5%) received target therapy and other 2 patients (4.5%) received best supportive care. 27.3% (12/44) patients received secondary cytoreduction surgery (SCS). After subsequent chemotherapy, 14 patients received PARPi retreatment as maintenance therapy. In patients who received platinum-based regimens (*n* = 35), 23 of 35 patients (65.7%) had complete/partial response (CR/PR), 8 of 35 (22.9%) had stable disease (SD), and 4 of 35 (12.1%) had progressive disease (PD). The ORR and DCR of patients who received subsequent chemotherapy was 65.7% and 88.6%, respectively. 15 patients (57.7%, 15/26) were reported to be platinum resistant with a platinum-free interval (PFI)  of < 6 months in patients whose platinum sensitivity of the second line platinum-based regimens was evaluable. Patients who received SCS after PARPi resistant associated with a borderline better PFS2 (median PFS2: 41.9 vs. 29.2 months, *P* = 0.051) and a non-significantly increased PFS2-PFS1 (median PFS2-PFS1: 12.2 vs. 9.8 months, *P* = 0.551). Patients with a PFI ≥ 12 months had a significantly better PFS2 (median PFS2: 37.0 vs. 25.3 months, *P* < 0.001) and a tendency towards a better PFS2-PFS1 than those with a PFI < 12 months (median PFS2-PFS1: 11.2 vs. 8.5 months, *P* = 0.334). A better PFS2 was observed in patients who received second PARPi maintenance therapy (median PFS2 of 35.4 vs. 28.8 months); however, the difference was not statistically significant (*P* = 0.200). A better PFS2-PFS1 was observed in patients who received second PARPi maintenance therapy (median PFS2-PFS1: 13.6 vs. 8.9 months, *P* = 0.002) than those without.

**Conclusions:**

In summary, some degree of resistance to standard subsequent platinum and non-platinum chemotherapy is noted in the entire cohort. A trend towards higher benefit from subsequent chemotherapy after first-line PARP inhibitors progression was observed in the PFI ≥ 12 months subgroup than those with PFI < 12 months. PARPi retreatment as maintenance therapy and SCS can be offered to some patients with PARPi resistance.

**Supplementary Information:**

The online version contains supplementary material available at 10.1186/s13048-024-01400-9.

## Introduction

Epithelial ovarian cancer is a major cause of death in women worldwide [1]. In 2016, there were approximately 57,200 new cases of ovarian cancer diagnosed and 27,200 ovarian cancer deaths in China [2]. Poly (adenosine diphosphate [ADP]–ribose) (PARP) inhibitors were the first approved cancer drugs that specifically targeted the DNA damage response in BRCA1/2 mutated breast and ovarian cancers. Compared with sporadic epithelial ovarian cancer (EOC) patients, both BRCA1 and BRCA2 associated patients have improved outcomes after primary therapy, including chemotherapy [3, 4]. Patients with germline mutations in BRCA1 or BRCA2, are extremely vulnerable to PARP inhibition. Clinical trials have demonstrated promising response rates among patients receiving PARP inhibitors (PARPi), especially for BRCA1 or BRCA2 mutation carriers [5–17]. 

The use of PARPi is growing widely as Food and Drug Administration (FDA) and National Medical Products Administration (NMPA) approvals have shifted its use from the recurrence setting to the frontline setting. But the majority will inevitably develop resistance. Preclinical and clinical data have revealed multiple mechanisms of PARPi resistance. The best clinically documented mechanism of resistance to PARP inhibitors is the presence of a BRCA reversion mutation which restore BRCA1/2 function. Mechanisms of PARPi resistance may have implications for post-progression therapies.

Currently, the subsequent management and chemotherapy response after first line PARPi progression is not fully elucidated in Chinese ovarian cancer population. This study aims to evaluating the subsequent management and outcomes after first line PARPi progression in ovarian cancer patients.

## Methods

### Study design

Following institutional review board approval, we performed a retrospective analysis of all ovarian cancer who progressed after first-line PARPi maintenance treatment between 2016/01 and 2021/9 in the department of gynecological oncology of cancer hospital, Chinese academy of medical sciences, national cancer center.

### Setting

Only patients with a diagnosis of epithelial ovarian cancer confirmed by an experienced gynecologic pathologist in our hospital were included. The patients’ full medical records were included in this study. Clinical and pathologic variables, treatment modalities, and outcomes were assessed. Stage was retrospectively assigned using the International Federation of Gynecology and Obstetrics (FIGO) 2014 staging system for ovarian cancer.

Next-generation sequencing (NGS) for the entire coding regions and exon/intron boundaries of the homologous recombination repair (HRR) genes were conducted in all patients included in our study. The applied method of mutation screening was described in detail in our previous report [16]. Only those pathogenic variants that lead to a truncated protein or have been previously reported to be associated with disease were considered to be deleterious.

### Variables

Clinical and pathologic variables included age at diagnosis, tumor size, primary tumor location, histology, FIGO stage, postoperative residual disease status, HRR status, first recurrence status, response to subsequent platinum-based chemotherapy.

### Sample size

A total of 124 patients received PARPi maintenance treatment after first-line chemotherapy during the study period in our center. 44 of them (35.5%) experienced a recurrence.

### Quantitative variables

The objective response rate (ORR) and disease control rate (DCR) parameters were evaluated to determine the response to subsequent chemotherapy. For the survival analyses, progression-free survival 1 (PFS1) was defined as the time from the date of diagnosis to the earlier date of assessment of progression or death from any cause in the absence of progression. PFS2 was defined as the time from the date of diagnosis to the earlier date of assessment of progression on the next anticancer therapy following study treatment or death from any cause. Overall survival (OS) was defined as the time from the date of diagnosis to death for which ovarian cancer was the primary or underlying cause. PFS2 − PFS1 was also explored in our study which was defined as time intervals from PFS1 to PFS2.

### Statistical analyses

Survival was estimated using the Kaplan–Meier product-limit method, and differences were tested for statistical significance using the log-rank test. Two-sided P values less than 0.05 were considered to be statistically significant. All analyses were performed using the SPSS Statistics 20.0 software.

## Results

### Study patients

Among these 44 recurrent patients, 17 of them (38.6%) had HRR gene mutation included: 18.2% (8 of 44) for BRCA1, 9.1% (4 of 44) for BRCA2 and 11.4% (5 of 44) for non-BRCA HRR genes (Table 1).

**Table 1:** Clinicopathological characteristics of patients in the entire cohort

Clinical Characteristics	n	%
**N**	44	
**Median age (Range)**	55 (29–76)	
**Age at diagnosis (y)**		
< 50	13	29.5
≥50	31	70.5
**Tumor size (cm)**		
<5	10	22.7
≥ 5	34	77.3
**Primary tumor location**		
Ovary	43	97.7
Fallopian tube	1	2.3
**Histology**		
Serous	43	97.7
Clear cell cancer	1	2.3
**FIGO Stage**		
I	0	0.0
II	1	2.3
III	31	70.5
IV	12	27.3
**Neoadjuvant chemotherapy**		
Yes	30	68.2
No	14	31.8
**Postoperative residual disease status**		
Optimal (R0/R1)	38	86.4
Suboptimal (≥ R1)	6	13.6
**PARPi received**		
Fluzoparib	3	6.8
Olaprib	17	38.6
Niraparib	24	54.5
**HRR status**		
BRCA1 mt	8	18.2
BRCA2 mt	4	9.1
Non-BRCA HRR mt	5	11.4
WT	27	61.4
**First recurrence**		
Platinum-sensitive recurrence	37	84.1
Platinum-resistant recurrence	7	15.9

*Abbreviations* Mt, mutation; WT, wild-type; FIGO, International Federation of Gynecology and Obstetrics; NA, not available

### Clinicopathological characteristics

Patients median age at diagnosis was 55 years (range: 29–76 years). 70.5% (31/44) of them were diagnosed after 50 years (Table 1). The FIGO 2014 distribution by stage was: stage II in 1 patient (2.3%), stage III in 31 patients (70.5%) and stage IV in 12 patient (27.3%) (Table 1). 97.7% (43/44) patients had high-grade serous carcinoma. 30 patients (30/44, 68.2%) received neoadjuvant chemotherapy. 38 patients (38/44, 86.4%) who had received secondary cytoreduction surgery (SCS) achieved R0/R1 resection (Table 1).

### Response to PARPi

All patients received PARP inhibitor maintenance treatment after first-line chemotherapy. 54.5% (24/44) patients received niraparib, 38.6% (17/44) patients received olaparib, and 6.8% (3/44) patients received fluzoparib. The median duration of PARPi in these patients was 11.1 months (range: 1.2–75.1 months). The median duration of PARPi in patients with or without HRR mutation was 12.0 months (range: 3.1–75.1 months) and 9.7 months (range: 1.2–26.9 months), respectively.

### Subsequent management

A total of 40 patients (40/44, 90.9%) received subsequent chemotherapy with 35 (35/44, 79.5%) and 5 (5/44, 11.4%) patients received platinum-based and non- platinum-based chemotherapy in our center (Table 2). 2 patients (4.5%) received target therapy including olaparib + apatinib and niraparib treatment. And other 2 patients (4.5%) received best supportive care (Table 2). 27.3% (12/44) patients received SCS.

**Table 2:** Details of subsequent therapy among the whole cohort

	n	%
**Platinum-based chemotherapy**	35	79.5
Paclitaxel + Platinum	16	36.4
Albumin-bound Paclitaxel + Platinum	13	29.5
PLD + Platinum	6	13.6
**Non-platinum-based chemotherapy**	5	11.4
PLD	4	9.1
PLD + Albumin-bound Paclitaxel	1	2.3
**Target therapy**	2	4.5
Olaparib + Apatinib	1	2.3
Niraparib	1	2.3
**Best supportive care**	2	4.5
**Cytoreduction surgery**		
Yes	12	27.3
No	32	72.7

*Abbreviations* PLD, pegylated liposomal doxorubicin

In patients who received platinum-based regimens (*n* = 35), 16 patients (16/35, 45.7%) received paclitaxel + platinum regimen, 13 patients (13/35, 37.1%) received albumin-bound paclitaxel + platinum regimen, 6 patients (6/35, 17.1%) received pegylated liposomal doxorubicin (PLD) + platinum regimen (Table 2). In patients who received non-platinum-based chemotherapy regimen (*n* = 5), 4 (4/5, 80.0%) and 1 (1/5, 20.0%) patients received PLD or PLD + albumin-bound paclitaxel regimen, respectively (Table 2).

### Response to subsequent chemotherapy

In patients who received platinum-based regimens (*n* = 35), 23 of 35 patients (65.7%) had complete/partial response (CR/PR), 8 of 35 (22.9%) had stable disease (SD), and 4 of 35 (12.1%) had progressive disease (PD) (Table 3). The ORR and DCR of patients who received subsequent chemotherapy was 65.7% and 88.6%, respectively. The platinum sensitivity of the second line platinum-based regimens was evaluable in 26 patients. Of these patients, 15 patients (57.7%, 15/26) were reported to be platinum resistant with a platinum-free interval of < 6 months.

**Table 3:** Response to subsequent platinum-based chemotherapy

Response	n	%
Complete response	5	14.3
Partial response	18	51.4
Stable disease	8	22.9
Progressive diesase	4	11.4
Total	35	

In patients who received non-platinum-based regimens (*n* = 5), 1 of 5 (20.0%) had stable disease (SD), and 4 of 5 (80.0%) had progressive disease (PD).

### PARPi after PARPi

After subsequent chemotherapy, 14 patients received PARPi retreatment as maintenance therapy with 13 patients received platinum-based chemotherapy previously (Table 4). Among these patients, 57.1% (8/14) patients received niraparib, 35.7% (5/14) patients received olaparib, and 7.1% (1/14) patients received fluzoparib (Table 4). At the date of data cutoff, 3 patients (21.4%) continued to receive PARPi treatment. The median duration of PARPi1 and PARPi2 was 11.9 months (range: 5.2–20.3 months) and 6.0 months (range: 1.0-15.4 months), respectively. Most patients (11/14, 78.6%) had a longer duration of PARPi1 than PARPi2. Type of PARPi, therapy duration, reasons for treatment discontinuation, PFS1 and PFS2 among patients who received PARPi retreatment as maintenance therapy were listed in Table 5.

**Table 4:** Clinicopathological characteristics of patients who received PARPi retreatment

Clinical Characteristics	n	%
**N**	14	
**Median age (Range)**	51.5 (38–76)	
**Age at diagnosis (y)**		
< 50	6	42.9
≥50	14	100.0
**Tumor size (cm)**		
<5	3	21.4
≥ 5	11	78.6
**Primary tumor location**		
Ovary	13	92.9
Fallopian tube	1	7.1
**Histology**		
Serous	14	100.0
Clear cell cancer	0	0.0
**FIGO Stage**		
III	10	71.4
IV	4	28.6
**Neoadjuvant chemotherapy**		
Yes	7	50.0
No	7	50.0
**Postoperative residual disease status**		
Optimal (R0/R1)	12	85.7
Suboptimal (≥ R1)	2	14.3
**PARPi 1 received**		
Fluzoparib	1	7.1
Olaprib	5	35.7
Niraparib	8	57.1
**2 L chemotherapy regimens**		
Platinum-based chemotherapy	13	92.9
Non-platinum-based chemotherapy	1	7.1
**PARPi 2 received**		
Fluzoparib	1	7.1
Olaprib	5	35.7
Niraparib	8	57.1
**HRR status**		
BRCA1 mt	1	7.1
BRCA2 mt	1	7.1
Non-BRCA HRR mt	2	14.3
WT	10	71.4
**First recurrence**		
Platinum-sensitive recurrence	14	100.0
Platinum-resistant recurrence	0	0.0
**Second recurrence**		
Platinum-sensitive recurrence	6	54.5
Platinum-resistant recurrence	5	45.5
NA	3	

*Abbreviations* Mt, mutation; WT, wild-type; FIGO, International Federation of Gynecology and Obstetrics; NA, not available

**Table 5:** Type of PARPi, therapy duration, reasons for treatment discontinuation, PFS1 and PFS2 among patients who received PARPi retreatment as maintenance therapy

ID	HRR status	Type of PARPi1	PFS1	Duration1	Type of PARPi2	PFS2	Duration2	reasons for treatment discontinuation	Duration1 > Duration2
1	wt	Fluzoparib	18.1	9.8	Niraparib	25.5	0.3	ongoing	Yes
2	wt	Niraparib	12.6	5.2	Olaparib	25.3	7.6	PD	No
3	wt	Niraparib	15.3	9.2	Olaparib	30.9	9.6	PD	No
4	wt	Niraparib	16.6	8.8	Olaparib	42.5	15.4	ongoing	No
5	wt	Niraparib	20.2	12.1	Niraparib	26.4	1.4	PD	Yes
6	wt	Niraparib	22.5	12.3	Fluzoparib	37.0	7.8	PD	Yes
7	non BRCA1/2 HRR mt	Niraparib	24.2	16.7	Olaparib	35.3	3.0	PD	Yes
8	wt	Niraparib	24.8	5.3	Niraparib	35.4	3.4	PD	Yes
9	wt	Niraparib	28.4	20.3	Niraparib	41.2	6.7	ongoing	Yes
10	BRCA1 mt	Olaparib	18.8	12.0	Niraparib	31.2	4.6	PD	Yes
11	wt	Olaparib	18.9	11.9	Niraparib	39.9	5.3	PD	Yes
12	non BRCA1/2 HRR mt	Olaparib	19.6	10.1	Niraparib	29.3	4.9	PD	Yes
13	wt	Olaparib	22.5	16.3	Olaparib	41.9	10.5	PD	Yes
14	BRCA2 mt	Olaparib	31.6	19.3	Niraparib	45.2	8.2	PD	Yes

*Abbreviations* Mt, mutation; WT, wild-type; PD, progressive disease; PFS, progression-free survival; HRR, homologous recombination repair

### Survival

The median follow-up was 36.5 months (range: 13.0–90.0 months). 7 patients (7/43, 15.9%) died during follow up. Median OS had not reached. 84.1% patients (37/44) were classified as platinum-sensitive recurrence, and 7 patients (15.9%) was classified as platinum-resistant recurrence. Median PFS for the entire cohort was 18.8 months. No difference of median PFS was observed for patients with or without HRR gene mutations, which was 19.4 months and 18.1 months, respectively (*P* = 0.173; Supplementary Fig. 1).

**Fig. 1:**
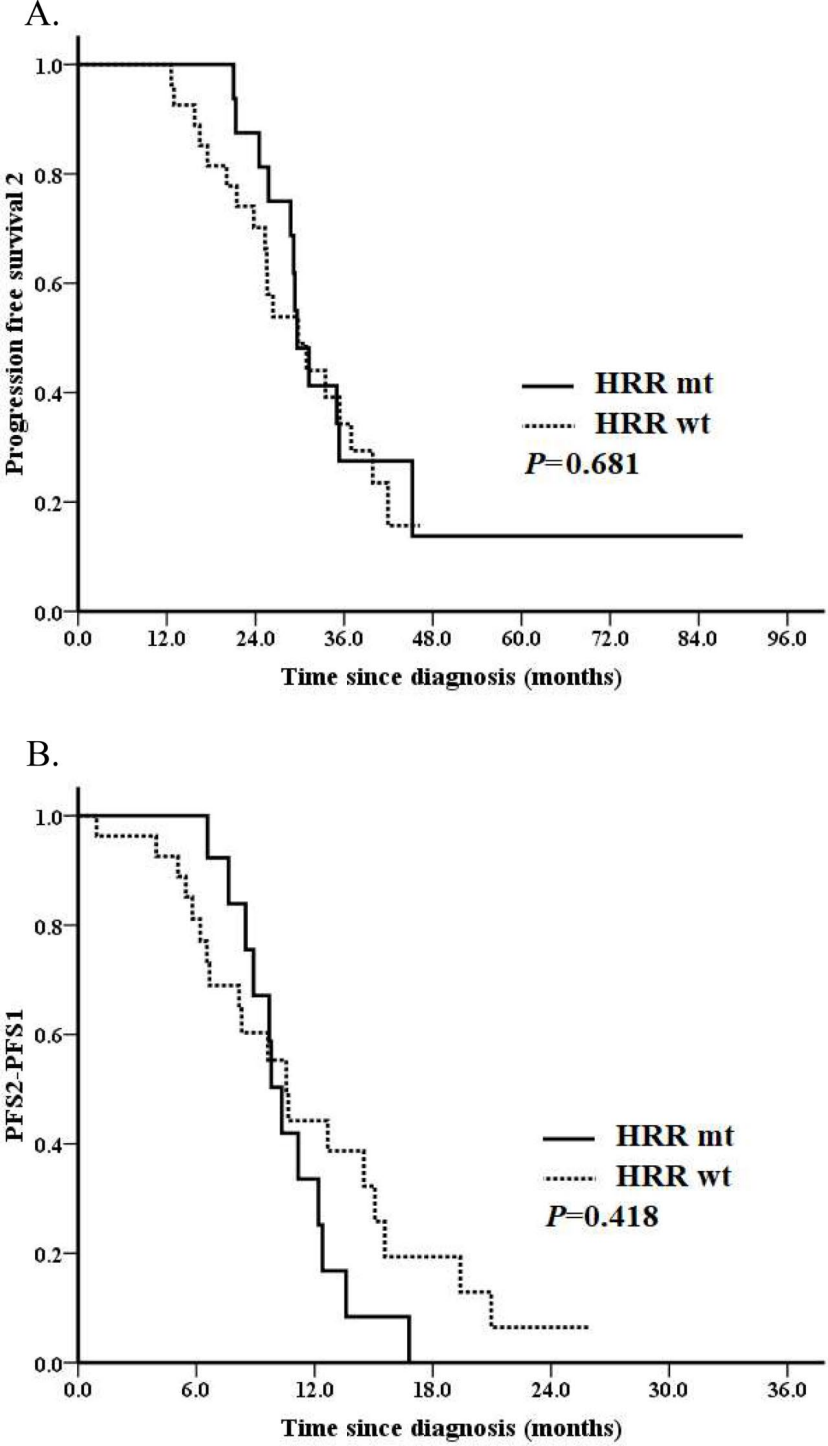
Survival analyses by the Kaplan–Meier method according to HRR gene mutation status in the entire cohort (*n* = 44). (**A**) Progression-free survival 2 (PFS2) and (**B**) PFS2 – PFS1

**Fig. 2:**
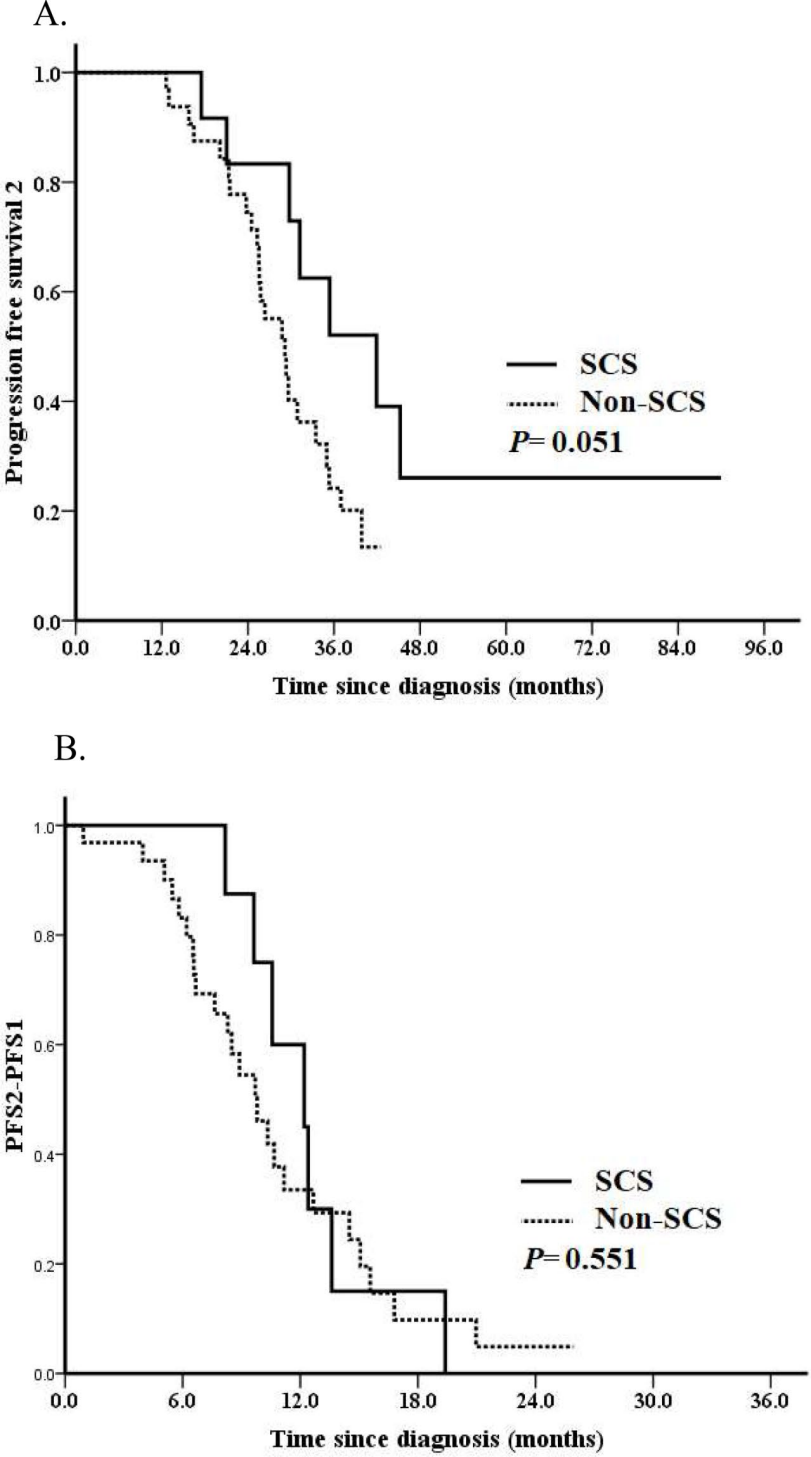
Survival analyses by the Kaplan–Meier method according to whether or not had received cytoreduction surgery after PARPi resistant (*n* = 44). (**A**) Progression-free survival 2 (PFS2) and (**B**) PFS2 – PFS1

**Fig. 3:**
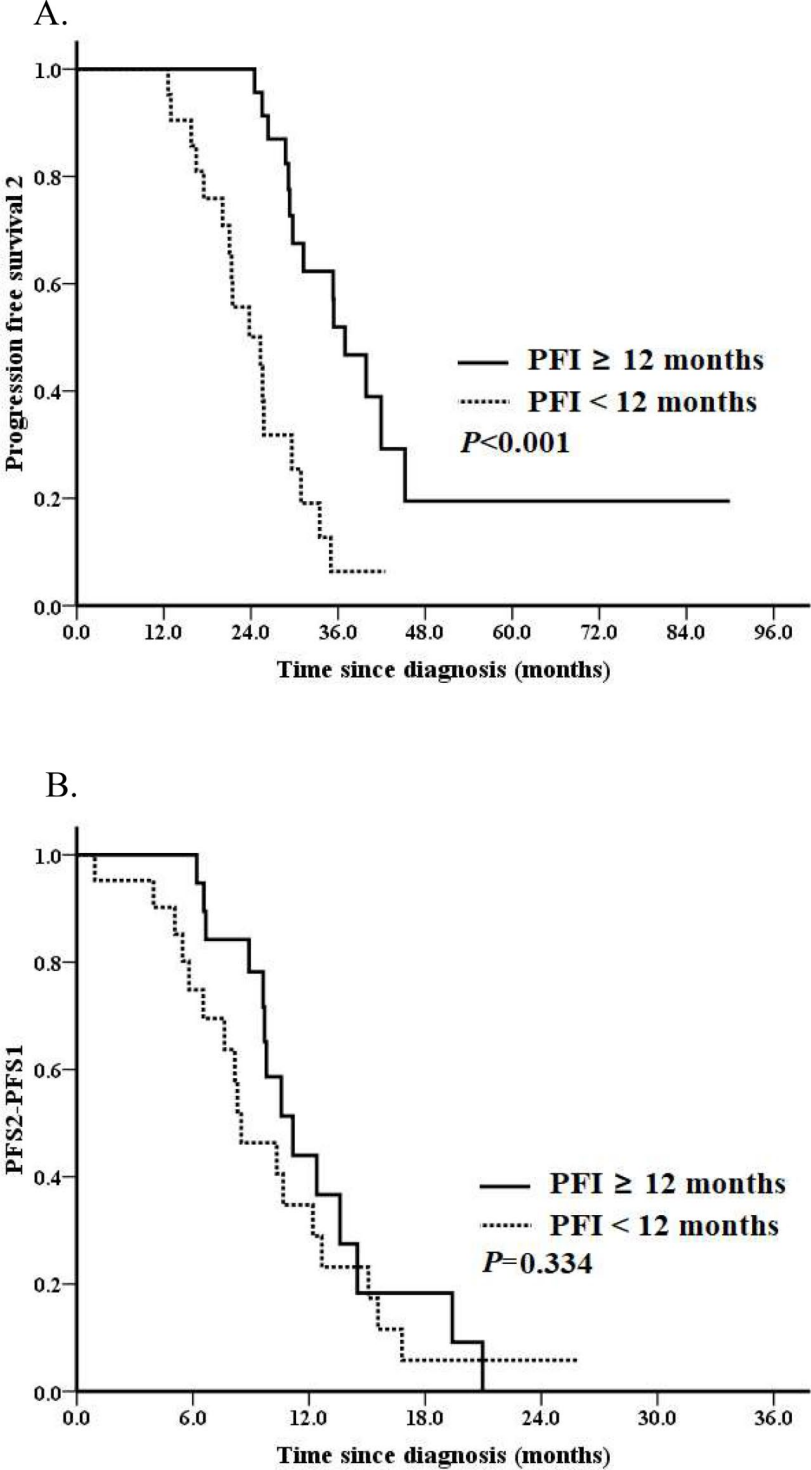
Survival analyses by the Kaplan–Meier method according to platinum free interval in the entire cohort (*n* = 44). (**A**) Progression-free survival 2 (PFS2) and (**B**) PFS2 – PFS1

**Fig. 4:**
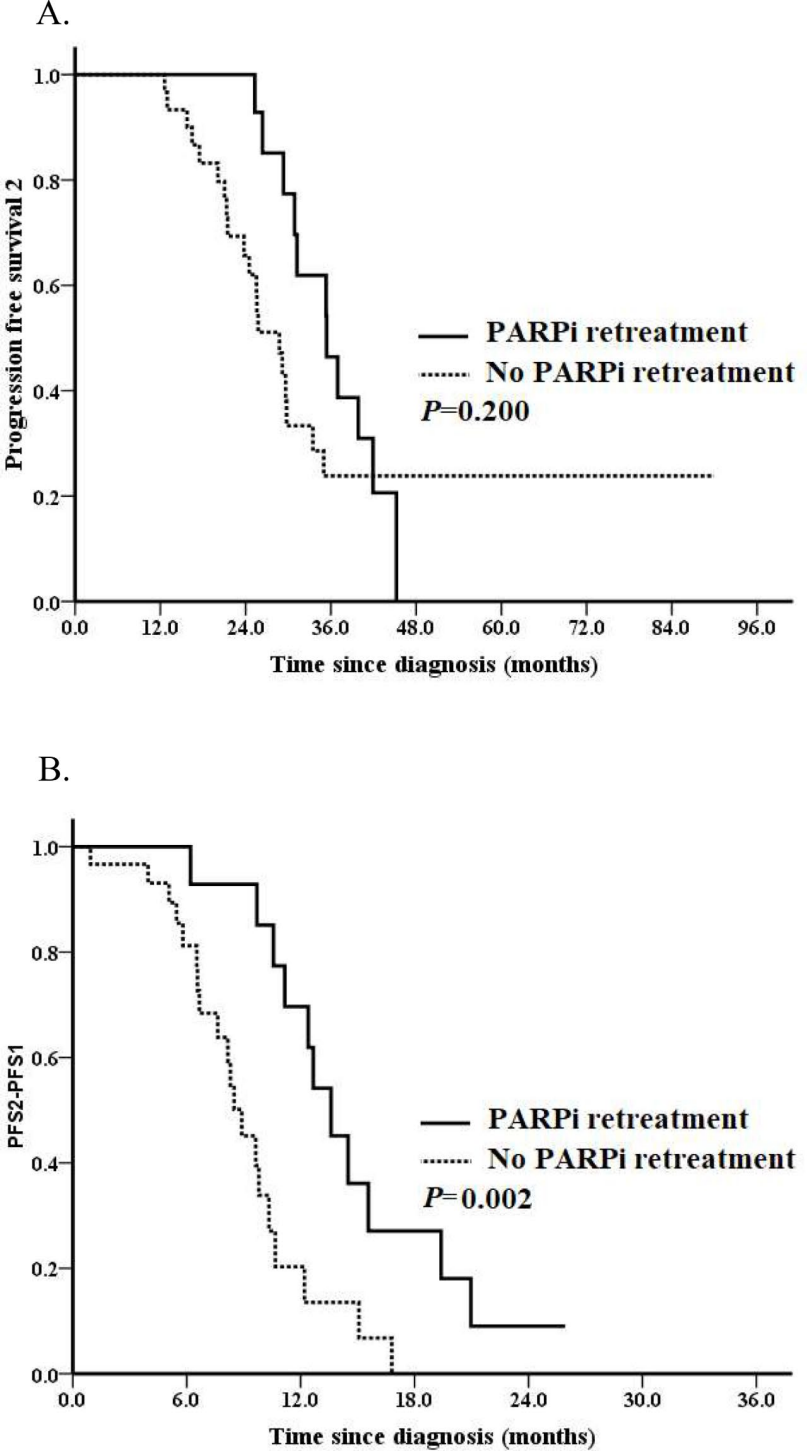
Survival analyses by the Kaplan–Meier method according to whether or not had received PARPi retreatment (*n* = 44). (**A**) Progression-free survival 2 (PFS2) and (**B**) PFS2 – PFS1

We further analyzed PFS2 in the entire cohort. 31 (70.5%) events occurred. Median PFS2 for the entire cohort was 29.8 months. No difference of median PFS2 was observed for patients with or without HRR gene mutations, which was 29.6 months and 29.8 months, respectively (*P* = 0.681; Fig. 1A). Patients who received SCS after PARPi resistant associated with a borderline better PFS2 (median PFS2: 41.9 vs. 29.2 months, *P* = 0.051; Fig. 2A). Patients with a PFI ≥ 12 months vs. < 12 months had a significantly better PFS2 (median PFS2: 37.0 vs. 25.3 months, *P* < 0.001; Fig. 3A). The median PFS2 for patients with a PFI < 6 months, 6–12 months, ≥ 12months was 16.5 months, 25.8 months and 37.0 months, respectively (*P* < 0.001). A better PFS2 was observed in patients who received second PARPi maintenance therapy (median PFS2 of 35.4 vs. 28.8 months) than those without; however, the difference was not statistically significant (*P* = 0.200; Fig. 4A).

Median PFS2-PFS1 for the entire cohort was 10.3 months. Median PFS2-PFS1 for patients with or without HRR mutation was 10.3 months and 10.6 months, respectively (*P* = 0.418; Fig. 1B). Patients who received SCS associated with a non-significantly increased PFS2-PFS1 (median PFS2-PFS1: 12.2 vs. 9.8 months, *P* = 0.551; Fig. 2B). Patients with a PFI ≥ 12 months had a tendency towards a better PFS2-PFS1 than those with a PFI < 12 months (median PFS2-PFS1: 11.2 vs. 8.5 months, *P* = 0.334; Fig. 3B). The median PFS2-PFS1 for patients with a PFI < 6 months, 6–12 months, ≥ 12months was 6.5 months, 10.3 months and 11.2 months, respectively (*P* = 0.009). A better PFS2-PFS1 was observed in patients who received second PARPi maintenance therapy (median PFS2-PFS1: 13.6 vs. 8.9 months, *P* = 0.002; Fig. 4B) than those without.

## Discussion

To our knowledge, the current study is one of the largest studies to investigate subsequent chemotherapy response and outcome of ovarian cancer after first-line PARPi progression to date. We found that some degree of resistance to standard subsequent platinum and non-platinum chemotherapy is noted in the entire cohort. Patients with PFI ≥ 12 months benefited more from subsequent chemotherapy than those with PFI < 12 months. PARPi retreatment and SCS can be offered to some patients with PARPi resistance.

Ovarian cancer is the commonest cause of death among gynaecological cancers. Clinical trials have demonstrated promising response rates among ovarian cancer patients with BRCA1/2 germline mutation receiving PARP inhibitors. However, durable responses of PARPi are uncommon and the development of acquired PARPi resistance often occurs during disease recurrence. All patients in our study developed PARPi resistance of first-line maintenance treatment after a median duration of 11.1 months (range: 1.2–75.1 months).

Emerging evidence showed that PARPi resistance could be correlated with induction of platinum resistance, due to overlapping resistance mechanism [18]. In the post-hoc comparison of SOLO2, some degree of resistance to standard subsequent platinum and non-platinum chemotherapy is noted in the olaparib arm [19]. However, many patients continue to see benefit from platinum chemotherapy after PARPi maintenance. Ang et al. found heavily pretreated ovarian cancer patients with BRCA1/2 mutation who are PARPi resistant retain the potential to respond to subsequent chemotherapy, including platinum-based agents [20]. Before the PARPi era, some randomized trials on platinum rechallenge in patients with recurrent EOC after more than 6 months from the last platinum cycle have shown response rates ranging from 47.2 to 66% [21]. In our study, the ORR and DCR of patients who received subsequent platinum chemotherapy was 65.7% and 88.6%, respectively. But, among patients whose platinum sensitivity of the second line platinum-based regimens was evaluable, more than half patients (57.7%, 15/26) were reported to be platinum resistant with a subsequent platinum-free interval of < 6 months.

Across diverse tumors and therapies, the treatment effect on PFS2 which indicated the effect of treatment beyond first progression correlated moderately with the treatment effect on OS [22]. PARPi maintenance treatment provided a PFS2 benefit and showed a long-term benefit in newly diagnosed ovarian cancer beyond first progression [23–25]. However, at the final analysis of SOLO3, PFS2 slightly favored olaparib, but the difference with chemotherapy was not statistically significant (median PFS2: 23.6 vs. 19.6 months, HR = 0.80; *P* = 0.229) [26]. The exact role of PARPi in maintenance and salvage treatment might be different.

A trend towards higher benefit from subsequent chemotherapy after PARPi resistant was observed in the PFI ≥ 12 months subgroup with 91.3% (21/23) of them received platinum-based chemotherapy retreatment in our study. We found a significantly longer median PFS2 and non-significantly PFS2-PFS1 for patients with a PFI ≥ 12 months than those with a PFI < 12 months. Rechallenge with subsequent platinum-based chemotherapy after PARPi maintenance should still be considered, but the efficiency in the PFI 6–12 subgroup warrants further research. Some studies found benefit from subsequent platinum chemotherapy after PARPi in the PFI 6–12 subgroup was poor and similar to benefit from chemotherapy in the non-platinum subgroup [27, 28]. 

Patterns of disease progression may be different in patients receiving PARPi or not. Cerda et al. found one-third of patients on PARPi maintenance experienced oligoprogression defined as limited to ≤ 3 sites and these patients may benefit from local consolidation therapy [29]. Cytoreduction surgery was an effective local therapy for oligoprogression. In the present study, 27.3% (12/44) patients SCS surgery after PARPi resistant. Patients who received SCS associated with a borderline better PFS2 and non-significantly improved PFS2-PFS1. In the forthcoming future, more patients will receive PARPi treatment as maintenance therapy or salvage treatment. The role of SCS in the era of PARPi maintenance should be defined precisely. A larger dataset is needed to validate these findings to assess if trials investigating local therapy for these patients is of value.

The opportunity for PARPi use after prior PARPi exposure exists. The OReO/ENGOT Ov-38 trial is the first to provide data on PARPi rechallenge in platinum sensitive recurrent ovarian cancer patients. The trial shows that maintenance olaparib provided a significant improvement in PFS vs. placebo, irrespective of BRCA mutation status, in non-mucinous platinum-sensitive relapsed ovarian cancer patients who had received one prior line of PARPi maintenance and were in response to their most recent platinum-based chemotherapy [30]. Real-life data support prospective evidence that patients with recurrent EOC may derive benefit of the re-treatment with PARPi in case of clear response to the last platinum-based therapy [31]. In the present study,14 patients (36.8%, 14/38) received PARPi maintenance treatment after subsequent chemotherapy. A better PFS2-PFS1 was found among these patients, although the median duration of PARPi2 was shorter than PARPi1. Among patients who received subsequent chemotherapy and achieved CR/PR (*n* = 23), PARPi retreatment associated with a non-significantly better PFS2-PFS1 (median: 13.6 vs. 9.8 months, *P =* 0.111). The exact role of PARPi after PARPi in epithelial ovarian cancer should be verified in more trials [32–34]. 

Acquired resistance to PARP inhibitors can develop via three general mechanisms: drug target-related effects, such as the upregulation of drug efflux pumps or mutations in PARP or functionally related proteins; restoration of HR owing to restoration of BRCA1/2 function; or loss of DNA end-protection and/or restoration of replication fork stability [35]. ARIEL4 is the first prospective report from a randomized trial demonstrating that the presence of a BRCA reversion mutation predicts for primary resistance to rucaparib [36]. Understanding the mechanisms of PARPi resistance, detecting them in real-time, such as through regular sampling by liquid biopsy, and optimizing targeted combinations, are critically needed [37]. Many combinatorial strategies aim to re-sensitize resistant cells to PARPi [32, 33]. However, most combinatorial strategies are thus far only in the preclinical or early-phase trial stages [38]. The best subsequent treatment choice may be determined according to the PARPi resistance mechanism in the near future.

There are two limitations to our study. The current study was retrospective, and the primary treatment was not assigned at randomized. All patients in this study came from our single center. Therefore, caution is required when interpreting our results. Studies with more patients and multi-center randomized controlled trials (RCTs) may confirm these results in the future. The best post-PARPi management should be studied in prospective manner.

## Conclusion

In summary, some degree of resistance to standard subsequent platinum and non-platinum chemotherapy is noted in the entire cohort. A trend towards higher benefit from subsequent chemotherapy after first-line PARP inhibitors progression was observed in the PFI ≥ 12 months subgroup than those with PFI < 12 months. PARPi retreatment as maintenance therapy and SCS can be offered to some patients with PARPi resistance.

### supplementary material

Below is the link to the electronic supplementary material.

Supplementary Material 1

## Data Availability

No datasets were generated or analysed during the current study.

## References

[1] Siegel RL, Miller KD, Fuchs HE, Jemal A (2021). Cancer statistics, 2021. CA Cancer J Clin.

[2] Zheng R, Zhang S, Zeng H, Wang S, Sun K, Chen R (2022). Cancer incidence and mortality in China, 2016. J Natl Cancer Cent.

[3] Vencken PMLH, Kriege M, Hoogwerf D, Beugelink S, van der Burg MEL, Hooning MJ (2011). Chemosensitivity and outcome of BRCA1- and BRCA2-associated ovarian cancer patients after first-line chemotherapy compared with sporadic ovarian cancer patients. Ann Oncol.

[4] Tan DSP, Rothermundt C, Thomas K, Bancroft E, Eeles R, Shanley S (2008). BRCAness syndrome in ovarian cancer: a case-control study describing the clinical features and outcome of patients with epithelial ovarian cancer associated with BRCA1 and BRCA2 mutations. J Clin Oncol.

[5] González-Martín A, Pothuri B, Vergote I, DePont Christensen R, Graybill W, Mirza MR (2019). Niraparib in patients with newly diagnosed Advanced Ovarian Cancer. N Engl J Med.

[6] Ray-Coquard I, Pautier P, Pignata S, Pérol D, González-Martín A, Berger R (2019). Olaparib plus Bevacizumab as First-Line maintenance in Ovarian Cancer. N Engl J Med.

[7] Coleman RL, Fleming GF, Brady MF, Swisher EM, Steffensen KD, Friedlander M (2019). Veliparib with First-Line Chemotherapy and as maintenance therapy in Ovarian Cancer. N Engl J Med.

[8] Del Campo JM, Matulonis UA, Malander S, Provencher D, Mahner S, Follana P (2019). Niraparib Maintenance Therapy in patients with recurrent ovarian Cancer after a partial response to the last platinum-based chemotherapy in the ENGOT-OV16/NOVA Trial. J Clin Oncol.

[9] Moore KN, Secord AA, Geller MA, Miller DS, Cloven N, Fleming GF (2019). Niraparib monotherapy for late-line treatment of ovarian cancer (QUADRA): a multicentre, open-label, single-arm, phase 2 trial. Lancet Oncol.

[10] Swisher EM, Lin KK, Oza AM, Scott CL, Giordano H, Sun J (2017). Rucaparib in relapsed, platinum-sensitive high-grade ovarian carcinoma (ARIEL2 part 1): an international, multicentre, open-label, phase 2 trial. Lancet Oncol.

[11] Coleman RL, Oza AM, Lorusso D, Aghajanian C, Oaknin A, Dean A (2017). Rucaparib maintenance treatment for recurrent ovarian carcinoma after response to platinum therapy (ARIEL3): a randomised, double-blind, placebo-controlled, phase 3 trial. Lancet.

[12] Moore K, Colombo N, Scambia G, Kim B-G, Oaknin A, Friedlander M (2018). Maintenance Olaparib in patients with newly diagnosed Advanced Ovarian Cancer. N Engl J Med.

[13] Pujade-Lauraine E, Ledermann JA, Selle F, Gebski V, Penson RT, Oza AM (2017). Olaparib tablets as maintenance therapy in patients with platinum-sensitive, relapsed ovarian cancer and a BRCA1/2 mutation (SOLO2/ENGOT-Ov21): a double-blind, randomised, placebo-controlled, phase 3 trial. Lancet Oncol.

[14] Penson RT, Valencia RV, Cibula D, Colombo N, Leath CA, Bidziński M (2020). Olaparib Versus Nonplatinum Chemotherapy in patients with platinum-sensitive relapsed ovarian Cancer and a germline BRCA1/2 mutation (SOLO3): a Randomized Phase III Trial. J Clin Oncol.

[15] Ray-Coquard I, Leary A, Pignata S, Cropet C, González-Martín A, Marth C (2023). Olaparib plus Bevacizumab first-line maintenance in ovarian cancer: final overall survival results from the PAOLA-1/ENGOT-ov25 trial. Ann Oncol.

[16] Li N, Zhu J, Yin R, Wang J, Pan L, Kong B et al. Treatment with Niraparib Maintenance Therapy in patients with newly diagnosed Advanced Ovarian Cancer: a phase 3 Randomized Clinical Trial. JAMA Oncol. 2023;e232283. 10.1001/jamaoncol.2023.2283 PMC10346505 37440217

[17] DiSilvestro P, Banerjee S, Colombo N, Scambia G, Kim B-G, Oaknin A (2023). Overall survival with maintenance olaparib at a 7-Year Follow-Up in patients with newly diagnosed Advanced Ovarian Cancer and a BRCA mutation: the SOLO1/GOG 3004 Trial. J Clin Oncol.

[18] Park J, Kim SI, Jeong SY, Kim Y, Bookman MA, Kim J-W (2022). Second-line olaparib maintenance therapy is associated with poor response to subsequent chemotherapy in BRCA1/2-mutated epithelial ovarian cancer: a multicentre retrospective study. Gynecol Oncol.

[19] Frenel JS, Kim JW, Aryal N, Asher R, Berton D, Vidal L et al. Efficacy of subsequent chemotherapy for patients with BRCA1/2-mutated recurrent epithelial ovarian cancer progressing on olaparib versus placebo maintenance: post-hoc analyses of the SOLO2/ENGOT Ov-21 trial. Ann Oncol. 2022;S0923-7534(22)01740-9. 10.1016/j.annonc.2022.06.011 35772665

[20] Ang JE, Gourley C, Powell CB, High H, Shapira-Frommer R, Castonguay V (2013). Efficacy of chemotherapy in BRCA1/2 mutation carrier ovarian cancer in the setting of PARP inhibitor resistance: a multi-institutional study. Clin Cancer Res.

[21] Gadducci A, Cosio S, Landoni F, Lissoni AA, Zola P, Laudani ME (2022). Response to Chemotherapy and clinical outcome of patients with recurrent epithelial ovarian Cancer after PARP inhibitor maintenance treatment: a Multicenter Retrospective Italian Study. Anticancer Res.

[22] Woodford RG, Zhou DD-X, Kok P-S, Lord SJ, Friedlander M, Marschner IC (2022). The validity of progression-free survival 2 as a surrogate trial end point for overall survival. Cancer.

[23] Oaknin A, Moore K, Colombo N, Scambia G, Kim B-G, Friedlander M (2019). Time to second progression (PFS2) and second subsequent therapy (TSST) for patients (pts) with newly diagnosed, advanced ovarian cancer (OC) and a BRCA mutation (BRCAm) treated with maintenance (mt) olaparib (ola): phase III SOLO1 trial. Ann Oncol.

[24] Han SN, Monk BJ, Gonzalez-Martin A (2020). Time to first subsequent therapy (TFST) and progression-free survival 2 (PFS2) from the phase 3 randomized, double-blind PRIMA/ENGOT-OV26/GOG-3012 study in patients with newly diagnosed ovarian cancer. Gynecol Oncol.

[25] Pautier P, Harter P, Pisano C, Cropet C, Hernando Polo S, Berger R (2021). Progression-free survival (PFS) and second PFS (PFS2) by disease stage in patients (pts) with homologous recombination deficiency (HRD)-positive newly diagnosed advanced ovarian cancer receiving bevacizumab (bev) with olaparib/placebo maintenance in the phase III PAOLA-1/ENGOT-ov25 trial. JCO.

[26] Richard T, Penson RV, Valencia N, Colombo CA, Leath III. Final overall survival results from SOLO3: phase III trial assessing olaparib monotherapy versus non-platinum chemotherapy in heavily pretreated patients with germline BRCA1 and/or BRCA2-mutated platinum-sensitive relapsed ovarian cancer. SGO. 2022.

[27] Nakazawa H, Nagao S, Narita M, Shibutani T, Jimi T, Yano H (2022). Effect of prior olaparib maintenance therapy for platinum sensitive recurrent ovarian cancer on response to subsequent platinum-based chemotherapy. J Obstet Gynaecol Res.

[28] Salarich AP, García IT, Burdalo BP, Gil-Martin M, Rodriguez JMP, Planas CF et al. Real-world-data on platinum outcomes after parp inhibitors progression in high grade serous ovarian cancer patients. International Journal of Gynecologic Cancer [Internet]. 2020 [cited 2022 Jun 19];30. Available from: https://ijgc.bmj.com/content/30/Suppl_4/A60.2.

[29] Cerda VR, Lu D, Scott M, Kim KH, Rimel BJ, Kamrava M (2022). Evaluation of patterns of progression on poly (ADP-ribose) polymerase inhibitor (PARPi) maintenance in ovarian cancer: a cross-sectional study. Int J Gynecol Cancer.

[30] Pujade-Lauraine E (2021). Maintenance olaparib rechallenge in patients (pts) with ovarian carcinoma (OC) previously treated with a PARP inhibitor (PARPi): phase IIIb OReO/ENGOT Ov-38 trial. Ann Oncol.

[31] Moubarak M, Harter P, Ataseven B, Traut A, Welz J, Baert T (2022). Re-treatment with PARPi in patients with recurrent epithelial ovarian cancer: a single institutional experience. Gynecol Oncol Rep.

[32] McMullen M, Karakasis K, Loembe B, Dean E, Parr G, Oza AM (2020). DUETTE: a phase II randomized, multicenter study to investigate the efficacy and tolerability of a second maintenance treatment in patients with platinum-sensitive relapsed epithelial ovarian cancer, who have previously received poly(ADP-ribose) polymerase (PARP) inhibitor maintenance treatment. Int J Gynecol Cancer.

[33] Park J, Lim MC, Lee J-K, Jeong DH, Kim SI, Choi MC (2022). A single-arm, phase II study of niraparib and bevacizumab maintenance therapy in platinum-sensitive, recurrent ovarian cancer patients previously treated with a PARP inhibitor: Korean Gynecologic Oncology Group (KGOG 3056)/NIRVANA-R trial. J Gynecol Oncol.

[34] Essel KG, Behbakht K, Lai T, Hand L, Evans E, Dvorak J (2021). PARPi after PARPi in epithelial ovarian cancer. Gynecol Oncol Rep.

[35] Dias MP, Moser SC, Ganesan S, Jonkers J (2021). Understanding and overcoming resistance to PARP inhibitors in cancer therapy. Nat Rev Clin Oncol.

[36] Kristeleit R, Lisyanskaya A, Fedenko A, Dvorkin M, de Melo AC, Shparyk Y (2021). Rucaparib versus chemotherapy in patients with advanced, relapsed ovarian cancer and a deleterious BRCA mutation: efficacy and safety from ARIEL4, a randomized phase III study. Gynecol Oncol.

[37] Lee EK, Matulonis UA (2020). PARP Inhibitor Resistance Mechanisms and implications for Post-progression Combination therapies. Cancers (Basel).

[38] Lheureux S, Oaknin A, Garg S, Bruce JP, Madariaga A, Dhani NC (2020). EVOLVE: a Multicenter Open-label single-arm clinical and translational phase II trial of Cediranib Plus Olaparib for Ovarian Cancer after PARP Inhibition Progression. Clin Cancer Res.

