# Supplementary material for: Subsequent management and outcomes after first-line PARP inhibitors progression in ovarian cancer patients
Source: J Ovarian Res. 2024 Apr 1;17:70. doi: 10.1186/s13048-024-01400-9 (PMC10983760; doi:10.1186/s13048-024-01400-9)

**Supplementary Fig. 1.** Survival analyses by the Kaplan–Meier method according to HRR gene mutation status in the entire cohort (n = 44). (A) Progression-free survival (PFS).


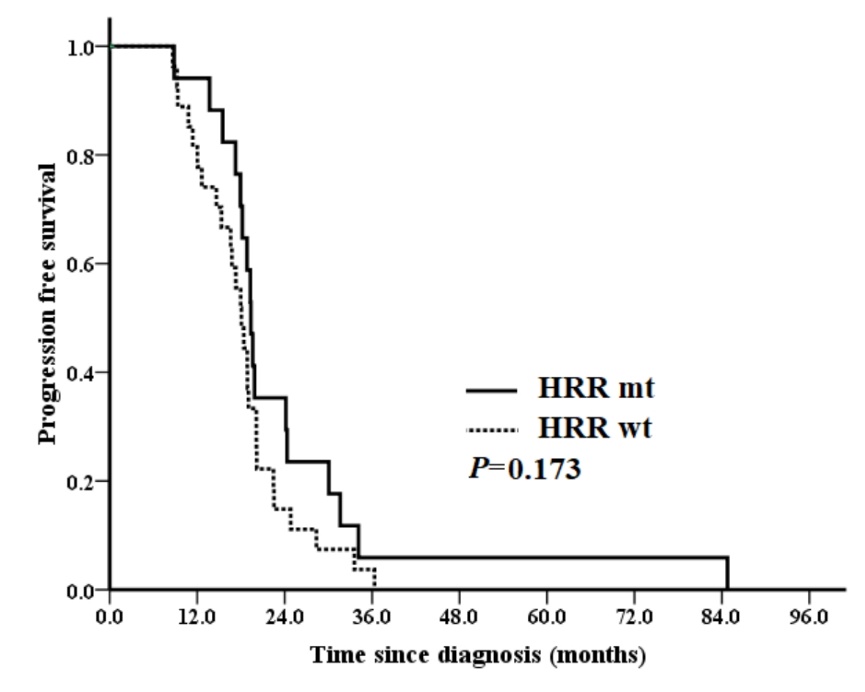

Supplement: Supplementary file 1 — Supplementary Material 1 [file 13048_2024_1400_MOESM1_ESM.docx]
